# The association between diet quality and chrononutritional patterns in young adults

**DOI:** 10.1007/s00394-024-03353-7

**Published:** 2024-02-22

**Authors:** Leanne Wang, Virginia Chan, Margaret Allman-Farinelli, Alyse Davies, Lyndal Wellard-Cole, Anna Rangan

**Affiliations:** 1 Discipline of Nutrition and Dietetics, Susan Wakil School of Nursing and Midwifery, Faculty of Medicine and Health, The University of Sydney, Sydney, NSW 2006 Australia; 2 Charles Perkins Centre, The University of Sydney, Sydney, NSW 2006 Australia; 3 Cancer Prevention and Advocacy Division, Cancer Council NSW, Sydney, NSW 2011 Australia

**Keywords:** Meal timing, Variability, Young adults, Diet quality, Evening eating, Chrononutrition

## Abstract

**Purpose:**

Young adults eat erratically and later in the day which may impact weight and cardiometabolic health. This cross-sectional study examined relationships between chrononutritional patterns and diet quality in two young adult populations: a university and community sample.

**Methods:**

Three days of dietary data were collected including food images captured using wearable cameras. Chrononutritional variables were extracted: time of first and last eating occasions, caloric midpoint (time at which 50% of daily energy was consumed), number of eating occasions per day, eating window, day-to-day variability of the above metrics, and evening eating (≥20:00h). The Healthy Eating Index for Australian Adults scored diet quality. Statistical analyses controlled for gender, body mass index, and socio-economic status.

**Results:**

No significant associations between chrononutritional patterns and diet quality were found for all participants (*n* = 95). However, differences in diet quality were found between university (*n* = 54) and community (*n* = 41) samples with average diet quality scores of 59.1 (SD 9.7) and 47.3 (SD 14.4), respectively. Of those who extended eating ≥20:00 h, university participants had better diet quality (62.9±SE 2.5 vs. 44.3±SE 2.3, *p* < 0.001) and discretionary scores (7.9±SE 0.9 vs. 1.6±SE 0.6, *p* < 0.001) than community participants. University participants consumed predominately healthful dinners and fruit ≥20:00h whereas community participants consumed predominately discretionary foods.

**Conclusion:**

For the general young adult population, meal timing needs to be considered. Food choices made by this cohort may be poorer during evenings when the desire for energy-dense nutrient-poor foods is stronger. However, meal timing may be less relevant for young adults who already engage in healthy dietary patterns.

**Supplementary Information:**

The online version contains supplementary material available at 10.1007/s00394-024-03353-7.

## Introduction

Despite continuous efforts to create healthier food environments, the obesity epidemic has worsened over the past decade with almost half of the young adult population in Australia now being affected by overweight and obesity [1]. Although genetic, environmental, and hormonal factors have an influence on body weight, diet, specifically excessive caloric intake, is the greatest contributor to weight gain [2]. In recent years, there has been a growing body of evidence in the area of chrononutrition which points towards the role of the timing of food intake on food choices [3, 4], caloric intake [3, 5] and its effects body weight [3, 5] and cardiometabolic health [5].

Young adults’ eating patterns are less structured [6–9] compared with older cohorts [10] with a substantial proportion of their energy intake occurring later in the day [8, 11]. Most published epidemiological studies suggest that such eating patterns have implications on body weight [3, 5] and cardiometabolic health [12–22] including increased blood pressure, poorer glycaemic control [5, 16], elevated high-sensitivity C-reactive protein [17], higher fatal cancer risk [23], and increased adiposity [12, 16]. Randomized crossover trials have found that a regular meal pattern where participants were provided six meals a day for 14 days had a beneficial impact on peak insulin and fasting total and low-density lipoprotein cholesterol levels when compared to an irregular meal pattern varying from three to nine meals a day within the same duration of time [22, 24]. However, it is unclear whether the effects of temporal eating patterns such as meal timing variability on weight and cardiometabolic health are independent of food choice, diet quality, and other dietary behaviours [19, 25].

Current literature examining the associations between temporal eating patterns and diet quality have had contrasting findings – whilst some suggest that grazing [9, 19] and late eating [3, 26, 27] are linked with lower diet quality, others suggest the opposite [27–29]. Most of these studies used nationally-representative data [9, 19, 26, 28, 29] and grazing was usually measured as the frequency of food consumption with [9, 19, 28] or without [26, 27, 29] consideration of the timing of these eating occasions. In our study, we aimed to explore how chrononutritional patterns such as meal timing variability and evening eating relate to diet quality. Our secondary aim was to compare these relationships between two groups of young adults. One group was a sample of university students and the other was a community sample who had eating behaviours that were more in line with the general Australian young adult population, that is, lower vegetable [30] and higher discretionary food consumption [31]. We hypothesised that individuals with greater meal timing variability and those who engaged in evening eating would have lower diet quality and that this relationship would be stronger in the community sample.

## Materials and methods

### Data collection

#### University sample

The university sample included students interested in their nutritional intake and quality. Interest in nutritional intake was ensured by offering participants feedback on their diet quality by Accredited Practising Dietitians as non-monetary incentive. Participants were recruited between October 2021 and May 2022 using methods such as word-of-mouth, including physical flyers around university campus, advertisements and invitations on university websites, and social media posts. To be eligible, participants had to be aged between 19 and 30 years inclusive, own a smartphone or digital camera, and must not have ever had an eating disorder or concerns about disordered eating. Participants completed a basic demographic questionnaire, providing information such as gender (female, male, other), height (cm), weight (kg), residential postcode, and highest degree or level of school completed (year 11 or below, year 12, Certificate III or IV, diploma or advanced diploma, bachelor’s degree, graduate certificate or graduate diploma, master’s degree or doctoral degree). Participants’ self-reported weight (kg) and height (cm) were used to calculate body mass index (BMI) (underweight < 18.5 kg/m^2^, healthy weight 18.5–24.9 kg/m^2^, overweight 25.0–29.9 kg/m^2^, obese ≥ 30.0 kg/m^2^), which has been found to be sufficiently accurate [32]. Socio-economic status was determined using the Socio-Economic Indexes for Areas 2016 Index of Relative Socio-economic Advantage and Disadvantage [33] based on participants’ postcodes. The top five deciles were labelled as high, and the bottom five deciles were labelled as low.

Each participant captured images of all foods and drinks consumed on three days using their smartphone. Images were captured immediately prior to the meal, snack or beverage so that the time stamp on the image reflected the start time of consumption. On the same three days, participants also kept a record of their intake on a commercial smartphone application (app) [34] or a paper-based food diary. On the app, participants recorded all foods and drinks consumed and amounts consumed. If a food or beverage they consumed was not on the app’s database, the closest substitute was used at the participant’s discretion. After completion, data on the app were emailed directly to researchers in a format that could readily be imported into the Foodworks Professional nutrient analysis software [35] and analysed using the Australian Food and Nutrient Database (AUSNUT 2011-13) [36, 37]. For participants who used a paper-based food diary, data were manually entered into the Foodworks Professional nutrient analysis software by researchers. This component of the study was approved by the University of Sydney Human Research Ethics Committee on the 9th of March 2022 (2021/513).

#### Community sample

The community sample included young adults from the sub-study (*n* = 133) [38] of a larger cross-sectional MYMeals project (*n* = 1001) [39]. Data from this group were collected in a similar manner to that of the university sample but used automated wearable cameras instead of user-initiated smartphone cameras and researcher-administered 24-hour recalls instead of food diaries recorded by participants.

Participants were aged 18–30 years, consumed foods or beverages prepared outside the home at least once a week, owned a smartphone, and were able to read and write English. Participants who were pregnant, lactating or had ever had an eating disorder were excluded. Recruitment and data collection methods have previously been described in the MYMeals study protocol [40]. In brief, participants in the sub-study wore an Autographer camera on a lanyard around the neck for all waking hours over three days. The camera automatically captured images from a first-person perspective every 30 s. On the same three days, they completed daily 24-hour dietary recall interviews with research dietitians via the Australian version of the Automated Self-Administered 24-hour recall Australia program [41]. Demographics were collected similarly to the university sample. Participants received an AUD $100 voucher as monetary incentive if they completed the study and returned the camera upon completion.

All images captured by the Autographer camera were coded by Accredited Practising Dietitians for the presence or absence of food and beverages, and matched with their 24-hour recall data. Two researchers independently matched the two sources (V.C. and A.D.) [42]. This component of the study was approved by the University of Sydney Human Research Ethics Committee on the 15th of July 2016 (2016/546).

### Data analysis

After the food images were matched with the food diaries and 24-hour recalls, data from participants with no unmatched main meals over the three days of data collection and no more than one unmatched snack or beverage per day were included for analysis. The data of all other participants were excluded.

The times of eating occasions were collated using the time and date stamps available from the food images. An eating occasion was defined as the consumption of any food or beverage with ≥210 kJ of energy [7]. Images captured within ≤ 15 min of each other were combined to form one eating occasion as per previous studies [7, 8] and the time of the latest image taken prior to food consumption was used to label the eating occasion. Each day was defined as the 24-hour period from 00:00 h to 23:59 h the same calendar day.

#### Chrononutritional variables

Eleven metrics measuring chrononutritional patterns were extracted – five eating pattern metrics, five meal timing variability metrics, and evening eating.

Eating pattern metrics including the chronological clock time of the first and last eating occasions, caloric midpoint, number of eating occasions per day, and eating window were extracted for all days and averaged over the three days of data collection. The caloric midpoint was defined as the time at which 50% of daily energy was consumed. The eating window was defined as the duration from the start time of the first eating occasion to the start time of the last eating occasion of the same day.

The variability of the above eating pattern metrics across the three days of data collection were used to measure meal timing variability: standard deviation of the first eating occasion (SD First), standard deviation of the last eating occasion (SD Last), standard deviation of the caloric midpoint (SD Caloric Midpoint), coefficient of variation of the daily number of eating occasions (CV Eating Occasions), and coefficient of variation of the eating window (CV Eating Window). These metrics were selected based on the methods of previous studies that used standard deviation to measure the variability of the first and last eating occasions and caloric midpoint [12, 16, 43] and coefficient of variation to measure the variability of the daily number of eating occasions and eating window [15]. The cut-offs used to categorize standard deviation and coefficient of variation scores as low, moderate, high, and very high variability were based on the difference in hours or number of eating occasions (Table 1). For example, participants with low variability in SD First varied in meal timing by less than two hours over the three days of data collection. An example of this is the consumption of the first meal or snack at 09:00 h on the first day, 10:00 h on the second day, and 10:45 h on the third day. These cut-offs were adapted from the methods of a previous study measuring meal timing variability in young adults [6].

**Table 1:** Cut-offs used to categorise meal timing variability metric scores as low, moderate, high, and very high variability and the equivalent difference in number of hours or eating occasions over three days

	SD First, Last and Caloric Midpoint		CV Eating Occasions		CV Eating Window	
	Cut-off	Hours	Cut-off	Number of Eating Occasions	Cut-off	Hours
Variability						
Low	< 1.00	< 2 h	< 10%	< 1 eating occasion	< 10%	< 2 h
Moderate	1.00-1.50	2-3 h	10–20%	1–2 eating occasions	10–20%	2-4 h
High	1.51-2.00	3-4 h	21–30%	2–3 eating occasions	21–30%	4-5 h
Very high	> 2.00	> 4 h	> 30%	> 3 eating occasions	> 30%	> 5

Evening eating was defined as continuing to eat at or after 20:00 h [44, 45], as determined using food image time stamps. Images with time stamps ≥20:00 h were further examined and labelled by food type as well as by food group, that is, predominately foods from the five food groups (grains, vegetables, fruits, dairy, lean meat and alternatives); or predominately discretionary, that is, foods and drinks that are high in saturated fat, added sugar, added salt, and/or alcohol that should only be consumed sometimes and in small amounts [46, 47]. If an eating occasion consisted of both five food group and discretionary foods, the one that provided more energy was used to label the eating occasion.

#### Diet quality

Diet quality was measured using the Healthy Eating Index for Australian Adults (HEIFA-2013), one of the best performing diet quality indices used in Australian adults [48] based on an inventory of diet quality indices construction criteria [49, 50]. It is a validated, gender-specific tool that assesses adherence to the Australian Dietary Guidelines [51]. The scoring system for this tool has been described elsewhere [52]. Briefly, the index consisted of 11 components: one for each of the five food groups; one for discretionary foods; four for specific nutrients (fatty acids, added sugar, sodium, alcohol), and one for water intake. Each component was scored a maximum of 10 points except for water intake and alcohol, which were scored a maximum of five. This totalled to an overall maximum score of 100. A higher score indicated a closer adherence to the dietary guidelines. For three of the five food group components (grains, vegetables, fruit), five of the 10 points were assigned to the adequate consumption of these food groups and the other five were assigned to the number of serves of wholegrains or how much variety was present in the types of fruit and vegetables consumed. For the fatty acid component, five of the 10 points were assigned to minimising saturated fat intake, and the other five were assigned to the adequate consumption of poly- and monounsaturated fats.

Within each of the 11 components, points were given incrementally for specified increases or decreases in number of serves consumed. Components where higher scores were given for a lower consumption were discretionary, saturated fat, sodium, added sugar, and alcohol. Increments and serves were different for each component and for different genders. For example, for the lean meat and alternatives component, males consuming ≥3.0 serves earned 10/10 points, 2.5 to < 3.0 serves earned 8/10, 2.0 to < 2.5 serves earned 6/10, 1.5 to < 2.0 serves earned 4/10, 1.0 to < 1.5 serves earned 2/10, and ≤0.5 earned 0/10. For females, ≥2.5 serves earned 10/10 points, 2.0 to < 2.5 serves earned 8/10, 1.5 to < 2.0 serves earned 6/10, 1.0 to < 1.5 serves earned 4/10, 0.5 to < 1.0 serve earned 2/10, and 0.0 serves earned 0/10. This scoring system was used on each day of the participants’ 24-hour recalls or food diaries via the Foodworks output and averaged across the three days of data collection to provide an overall diet quality score, as well as scores for individual diet quality components.

### Statistical analysis

Statistical analyses were conducted using SPSS software, v27.0 for Windows (IBM, Armonk, NY, USA) [53]. Descriptive statistics (frequency, mean, standard deviation, and percentage (%)) were used to summarise sample characteristics, chrononutritional variables, and diet quality. Differences between university and community groups in chrononutritional variables and diet quality were determined using the t-test for normally distributed data and the Mann-Whitney U test for non-normal data. Normality was determined using the Shapiro-Wilk test. Linear regression was used to identify associations between chrononutritional variables and diet quality, including overall diet quality and individual diet quality components (discretionary, total vegetable, total fruit). Univariate general linear models tested for differences in diet quality between participants who continued to eat at or after 20:00 h and participants who concluded eating by 20:00h, as well as between university and community participants who continued to eat at or after 20:00h. In the linear regression and univariate general linear model, analyses were adjusted for gender, BMI, and socio-economic status. A *p* value of ≤ 0.05 was considered statistically significant.

## Results

A total of 95 participants were included in the analysis; 54 in the university sample and 41 in the community sample. Participant characteristics can be found in Table 2. A total of 1411 eating occasions across 285 days were included for analysis.

**Table 2:** Characteristics of the university and community sample participants and all males and females

	University sample (*n* = 54)			Community sample (*n* = 41)		
	All	Female (*n* = 47)	Male (*n* = 7)	All	Female (*n* = 27)	Male (*n* = 14)
Body mass index (kg/m^2^)						
<18.5	8	7	1	0	0	0
≥18.5 < 25	42	36	6	28	20	8
≥25 < 30	2	2	0	7	3	4
≥30	2	2	0	6	4	2
Socio-economic status^a^						
Higher	47	40	7	22	14	8
Lower	4	4	0	19	13	6
Highest education attained						
Secondary school or less	0	0	0	15	10	5
Trade or diploma	2	1	1	7	7	0
University degree	52	46	6	19	10	9

^a^Socio-economic status was determined using the Socio-Economic Indexes for Areas 2016 Index of Relative Socio-economic Advantage and Disadvantage [33] based on postcode. The top five deciles were labelled as higher and the bottom five deciles were labelled as lower

Diet quality scores, and chrononutritional variables are summarised in Table 3. For the combined sample, the average overall diet quality score was 54.0, discretionary component score was 5.4/10, and fruit and vegetable component scores were both 2.8/5. All meal timing variability metrics scored high to very high except SD First and SD Last, which were moderate.

**Table 3:** Mean and standard deviation of overall and individual diet quality component scores and chrononutritional variables and differences between the university sample and the community sample

	All participants (*n* = 95)	University sample (*n* = 54)	Community sample (*n* = 41)	
	Mean ± SD	Mean ± SD	Mean ± SD	*p*
**Diet quality scores**				
Overall diet quality^a^	54.0 ± 13.3	59.1 ± 9.7	47.3 ± 14.4	< 0.001
Discretionary component^b^	5.4 ± 4.3	7.9 ± 3.3	2.3 ± 3.4	< 0.001
Total vegetable component^b^	2.8 ± 2.0	2.7 ± 1.5	2.9 ± 2.5	0.816
Total fruit component^b^	2.8 ± 2.7	2.4 ± 1.7	3.3 ± 3.6	0.937
**Chrononutritional variables**				
Time of first eating occasion (hh:mm)	10:05 ± 01:42	10:25 ± 01:46	09:39 ± 01:32	0.031
Time of last eating occasion (hh:mm)	20:17 ± 01:14	20:18 ± 01:09	20:17 ± 01:21	0.958
Caloric midpoint (hh:mm)	16:04 ± 02:02	15:58 ± 02:10	16:12 ± 01:50	0.568
Number of eating occasions per day	5.0 ± 1.4	4.4 ± 1.2	5.7 ± 1.3	< 0.001
Daily eating window (h)	10.2 ± 2.1	9.9 ± 2.1	10.6 ± 2.1	0.086
SD First (h)	1.3 ± 1.1	1.1 ± 0.8	1.4 ± 1.4	0.615
SD Last (h)	1.2 ± 0.9	1.2 ± 0.9	1.2 ± 0.9	0.934
SD Caloric Midpoint (h)	2.6 ± 1.4	2.7 ± 1.4	2.4 ± 1.5	0.207
CV Eating Occasions (%)	25.2 ± 16.3	24.1 ± 17.5	26.7 ± 14.7	0.276
CV Eating Window (%)	20.6 ± 19.3	20.2 ± 18.2	21.0 ± 20.9	0.886

Significance at *p* value ≤ 0.05

^a^Overall diet quality was scored out of 100 using the Healthy Eating Index for Australian Adults (HEIFA-2013)

^b^Each individual diet quality component was scored out of 10

Compared to the university sample, overall diet quality and discretionary component scores of the community sample were significantly lower by 11.8 and 5.6 points respectively. The time of the first eating occasion of the community sample was significantly earlier than that of the university sample by 46 min, and a significantly higher number of eating occasions were recorded in the community sample. No significant differences were found between the groups in any of the meal timing variability metrics.

**Fig. 1:**
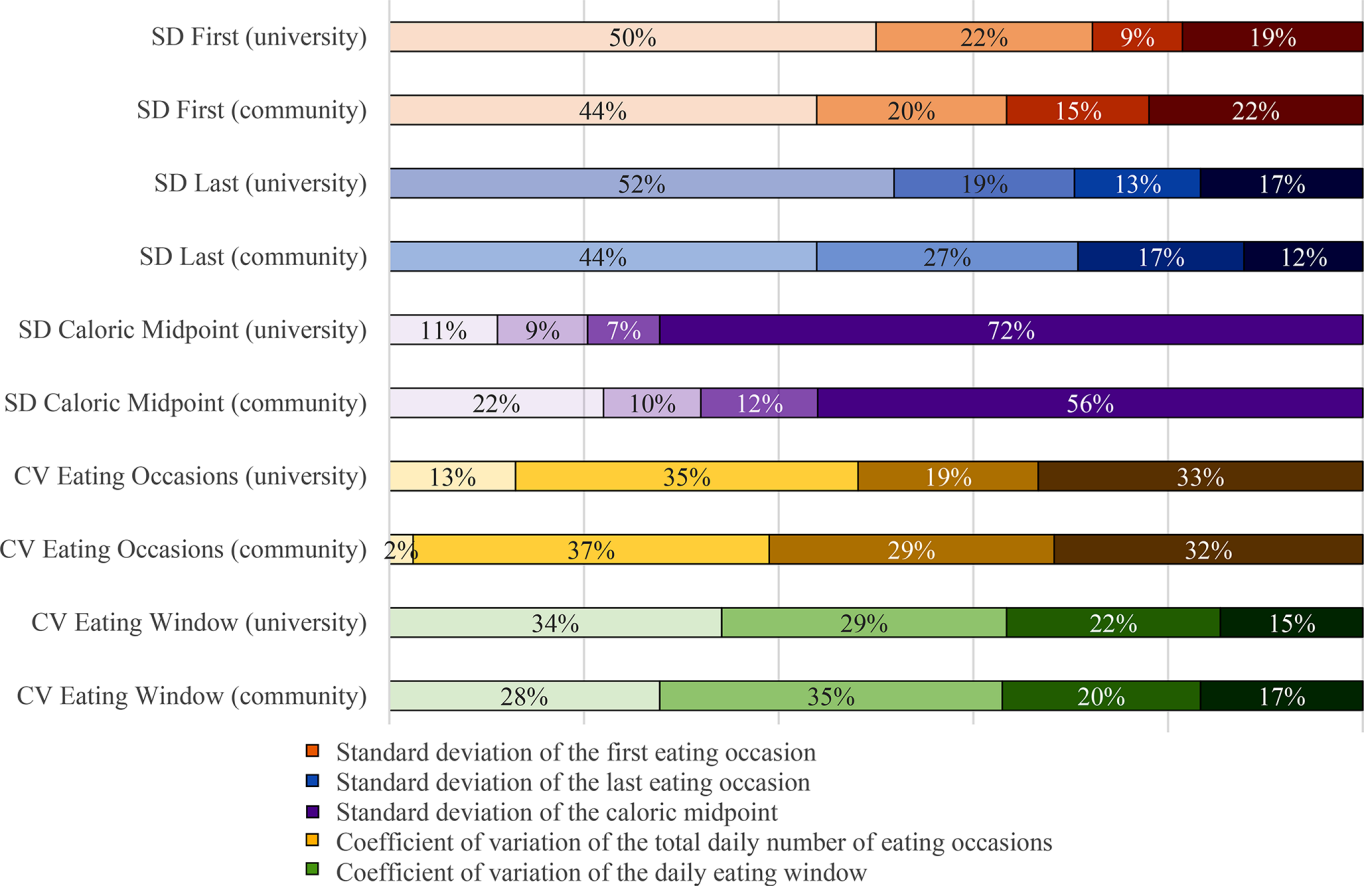
Stacked bar chart showing the percentage of participants in the university sample and community sample that fell into the low, moderate, high, and very high meal timing variability categories for each metric over the 3 days of data collection

Figure 1 shows the distribution of participants in each variability category for the meal timing variability metrics. The overall proportion of participants in each variability category was similar for both groups except for caloric midpoint – whilst 72% of the university group fell into the very high variability for the caloric midpoint category, only 56% of the community group fell into the very high variability category for this metric.

Table 4 shows the correlations between the chrononutritional variables (except for evening eating as this was a categorical variable) and diet quality for all participants after controlling for gender, BMI, and socio-economic status. Overall diet quality was not significantly correlated with any chrononutritional variable. However, some significant correlations were found between chrononutritional variables and individual diet quality components; for example, an earlier first eating occasion and a longer daily eating window were associated with higher fruit and vegetable scores and more discretionary food intake, and more eating occasions per day were associated with more discretionary food and vegetable intake. Further, there were no significant correlations between eating pattern and meal timing variability metrics with overall diet quality when the university and community groups were analysed separately (Table S1).

**Table 4:** Associations between chrononutritional variables and diet quality for all participants after adjusting for gender, body mass index, and socio-economic status^a^

	Overall Diet Quality		Discretionary Component		Total Vegetable Component		Total Fruit Component	
	β	*p*	β	*p*	β	*p*	β	*p*
Time of first eating occasion	0.086	0.908	0.863	< 0.001	-0.260	0.031	-0.334	0.047
Time of last eating occasion	-0.366	0.731	-0.267	0.432	0.090	0.608	0.157	0.517
Caloric midpoint	-0.493	0.441	-0.028	0.892	-0.038	0.717	-0.042	0.773
Number of eating occasions per day	-1.067	0.236	-1.528	< 0.001	0.471	0.001	0.410	0.043
Daily eating window	-0.181	0.769	-0.679	< 0.001	0.207	0.037	0.280	0.043
SD First	-1.967	0.090	-0.155	0.678	-0.125	0.512	0.144	0.588
SD Last	0.095	0.949	0.745	0.114	-0.458	0.057	0.169	0.617
SD Caloric Midpoint	0.237	0.787	0.335	0.231	0.178	0.213	-0.101	0.611
CV Eating Occasions	0.135	0.986	3.654	0.142	-0.371	0.772	-0.264	0.882
CV Eating Window	-2.111	0.749	3.713	0.075	-1.739	0.105	0.496	0.741

Significance at *p* value ≤ 0.05.^a^Socio-economic status was determined using the Socio-Economic Indexes for Areas 2016 Index of Relative Socio-economic Advantage and Disadvantage [33] based on their postcode

### Evening eating

Table 5 shows differences in diet quality between participants who continued to eat at or after 20:00 h and those who had finished eating by that time. No significant differences in overall diet quality and individual diet quality component scores were found in the combined sample. However, significant differences were found in the university sample, where individuals who continued to eat at or after 20:00 h had significantly higher overall diet quality and fruit component scores than those who did not eat after 20:00 h.

**Table 5:** Differences in diet quality between participants who concluded eating by 20:00 h and participants who continued to eat at or after 20:00 h (after adjusting for gender, BMI, and socio-economic status)

	< 20:00 h Mean ± Std. error	≥20:00h^c^ Mean ± Std. error	p value
All participants	*n* = 16	*n* = 79	
Overall diet quality^a^	51.7 ± 3.3	51.4 ± 1.7	0.920
Discretionary component^b^	4.6 ± 1.1	4.1 ± 0.6	0.639
Total vegetable component^b^	2.5 ± 0.5	2.8 ± 0.3	0.608
Fruit component^b^	2.3 ± 0.8	2.9 ± 0.4	0.487
University sample	*n* = 11	*n* = 43	
Overall diet quality^a^	54.6 ± 3.4	62.9 ± 2.5	0.010
Discretionary component^b^	6.5 ± 1.3	7.9 ± 0.9	0.230
Total vegetable component^b^	2.1 ± 0.6	2.6 ± 0.4	0.304
Fruit component^b^	1.5 ± 0.6	2.7 ± 0.5	0.046
Community sample	*n* = 5	*n* = 36	
Overall diet quality^a^	57.6 ± 6.3	44.3 ± 2.3	0.052
Discretionary component^b^	3.6 ± 1.5	1.6 ± 0.6	0.227
Total vegetable component^b^	3.1 ± 1.2	2.8 ± 0.5	0.842
Fruit component^b^	4.7 ± 1.7	3.0 ± 0.6	0.360

Significance at *p* value ≤ 0.05

^a^Overall diet quality was scored out of 100 using the Healthy Eating Index for Australian Adults (HEIFA-13)

^b^Each diet quality component was scored out of 10

^c^Includes participants with eating occasions at or after 20:00 h on at least one out of the three days of data collection

In addition, university participants who continued to eat at or after 20:00 h had significantly better diet quality (62.9 ± SE 2.5 vs. 44.3 ± SE 2.3, *p* < 0.001) and discretionary scores (7.9 ± SE 0.9 vs. 1.6 ± SE 0.6, *p* < 0.001) than community participants who ate continued to eat at or after 20:00 h when adjusted for gender, BMI, and socio-economic status.

As shown in Table 6, the majority of the food choices made by university participants in the evenings were from the five food groups, with a large proportion of this being healthful dinners and fruit or fruit products. In contrast, the majority of the food choices made by community participants in the evenings were discretionary.

**Table 6:** Types of foods consumed at or after 20:00 h by the university sample and the community sample

Types of foods	University sample	Community sample
Five food group	*n* = 93	*n* = 56
	Dinner (predominately from the five food groups) (*n* = 51) Fresh or fruit products (*n* = 23) Dairy products (*n* = 11) Cereals (*n* = 4) Vegetables (*n* = 2) Nuts (*n* = 2)	Dinner (predominately from the five food groups) (*n* = 28) Dairy products (*n* = 13) Fruit or fruit products (*n* = 11) Meat or fish (*n* = 2) Vegetables (*n* = 1) Nuts (*n* = 1)
Discretionary	*n* = 41	*n* = 65
	Chocolate (*n* = 13) Dinner (predominately discretionary) (*n* = 7) Ice cream and frozen desserts (*n* = 5) Cookies and biscuits (*n* = 4) Cakes and pastries (*n* = 3) Alcoholic beverages (*n* = 3) Artificially sweetened beverages (*n* = 2) Confectionary (*n* = 2) Popcorn (*n* = 1) Sugar-sweetened beverages (*n* = 1)	Cakes and pastries (*n* = 14) Alcoholic beverages (*n* = 10) Sugar-sweetened beverages (*n* = 10) Dinner (predominately discretionary) (*n* = 8) Cookies and biscuits (*n* = 8) Ice cream and frozen desserts (*n* = 6) Chocolate (*n* = 5) Popcorn (*n* = 1) Chips (*n* = 1) Confectionary (*n* = 1) Protein bars (*n* = 1)

## Discussion

The primary aim of this study was to examine the associations between chrononutritional patterns and diet quality in two groups of young adults. Chrononutritional variables included eating pattern metrics, meal timing variability metrics, and evening eating. Contrary to our initial hypothesis, there were no significant associations between any of the chrononutritional variables and overall diet quality, although some chrononutritional variables were significantly correlated with individual diet quality components. For example, an earlier first eating occasion and a longer daily eating window were associated with higher fruit and vegetable scores and more discretionary food intake. More eating occasions per day was also associated with more discretionary food and vegetable intake. However, these associations are likely simply due to having more opportunities to consume foods and beverages, resulting in an increase in consumption of all food groups used to measure the individual diet quality components.

The secondary aim of the study was to compare chrononutritional variable and diet quality associations between the two groups of young adults. One was a university sample interested in their nutritional intake and the other was a community sample with eating behaviours more in line with the general Australian young adult population. There were no significant associations between any of the chononutritional variables and overall diet quality for either group except for their evening food choices. Whilst participants in the university sample had better diet quality than the community sample overall, those who continued to eat at or after 20:00 h saw further significant improvements to their diet quality. This improvement in diet quality was not seen in evening eaters from the community sample. This was due to the university group choosing predominately foods from the five food groups, in particular evening meals (dinner) and fruit, and less discretionary choices than the community sample in the evenings. This finding was also in contrast to our initial hypothesis.

Evenings are a time when the desire for energy-dense nutrient-poor foods is stronger [28, 54–56]. This may in part be due to factors related to having a late chronotype, a dominant form of sleep regimen for young adults [57], such as decreased self-control [58] or sleep deprivation [59]. Studies have shown that late night eating events were more likely to consist of discretionary snacks [60, 61] due to an increased desire for these foods despite lowest hunger levels at this time [55]. Systematic and scoping reviews examining the role of chronotype on meal timing and dietary intake have also found a link between late chronotypes and a high consumption of nutritionally poor or high fat foods [3], as well as the tendency to eat main meals later in the day compared to early or intermediate chronotypes [3, 62]. This can lead to adverse health outcomes such as weight gain and poorer glycemic control [5]. On the contrary, health-conscious individuals are likely to have greater nutrition knowledge that enable them to override the desire for energy-dense nutrient-poor foods and make more conscious food choices at night [63, 64]. Previous studies have shown that night-time food choices made by health-conscious individuals include nutrient-dense foods such as protein-rich beverages [65] or fruit [44]. This is consistent with the findings in our university sample. Sebastian et al. discovered six main late evening (≥20:00 h) eating patterns in US adults using cluster analysis – (1) baked goods, sandwiches, and other desserts, (2) mixed dishes and meats, (3) alcoholic beverages and savoury snacks, (4) nuts and candy, (5) fruit, and (6) milk and baked goods [44]. These eating patterns represented the food/beverage groups that contributed the most energy during late evening eating. They found that individuals following the fruit pattern had higher diet quality scores than individuals who did not engage in late evening eating as well as individuals with any of the other late evening eating patterns as it increased scores for total and whole fruit and moderated the scores for sodium, refined grains, and saturated fats [44]. Taken together, the literature confirms our findings and indicates a congruency between nutrition knowledge or interest level and healthy eating habits. Interventions geared towards modifying dietary behaviours later in the day such as evening food choices may be more successful at improving diet quality [4].

Whilst our study did not find any associations between the stability of meal timing and overall diet quality, the current literature around eating patterns and meal timing variability versus diet quality is inconclusive. Unstable meal times, defined as frequent eating occasions occurring at unconventional times throughout the day, was associated with lower diet quality scores in some studies [9, 19] whereas grazing, defined by the frequency of snack consumption, was associated with higher diet quality scores in other studies [27–29]. Some studies have suggested that the timing of grazing may play a role in diet quality – snacking in the morning increased diet quality whilst snacking in the evening decreased diet quality [26–28, 61, 66] due to the types of foods selected. The link between chronotype and meal regularity and timing, caloric midpoint, meal frequency, eating window, and timing of meals relative to sleep and wake times has also not yet been established due to the small number of studies that have explored this relationship [62].

A key strength of our study was the use of a combination of food images and food diaries to collect dietary data as this may help minimise inaccurate recalls and missing data [67]. Whilst the food images were able to provide objective data around the time of food consumption and enhance self-reported dietary intake by revealing unreported foods and misreporting errors [68–70], the food diaries were able to provide data that could not be determined from image analysis alone such as hidden ingredients [71–74]. Our paper compared meal timing variability over multiple days with diet quality. Most studies examining this relationship used the distribution of meals and snacks over one day [9, 19].

Our study had several limitations, the most notable being the small sample size which may have been insufficiently powered to detect differences between groups. Although the community sample was more diverse in gender, BMI, and socio-economic status than the university sample, their results are likely not generalisable to the Australian young adult population as they had a higher HEIFA score [52] and a lower proportion of individuals in the overweight and obese BMI categories [1] than the general young adult population in Australia. This is based on data from the 2011–2012 National Nutrition and Physical Activity Survey [52] and the Australian Bureau of Statistics [1]. They showed that the average HEIFA score for young Australian adults (18–24 years) was 41.6, 4.7 points lower than our community sample [52], and that the proportion of young people (18–24 years) whose weights were in the overweight and obese BMI categories in 2017-18 was 46%, 14% more than our community sample [1]. As with most dietary assessment methods, participants may have altered their eating behaviour on the days of data collection or chosen days that were more convenient to record such as at home or away from social settings. Therefore, collected dietary data may not be representative of participants’ usual intake and week-long cross-sectional studies are likely needed to capture habitual eating behaviours [43]. The three days of data collection was also different for the university and community samples as one was over consecutive days and the other was over non-consecutive days and so they may not be directly comparable. Dietary data collected over non-consecutive or random days have been shown to be more representative of usual intake [75, 76] likely because foods and amounts consumed on consecutive days are related, e.g., the consumption of leftovers or eating less as a result of excessive intake the previous day [77]. For future studies, larger sample sizes are needed to verify our findings and data on individuals’ chronotypes should be collected given its influence on eating patterns.

## Conclusion

Our study explored the relationship between chrononutritional patterns and diet quality and compared this relationship across two groups of young adults – a university sample with better diet quality and a community sample with eating behaviours more in line with the general Australian young adult population. Within the university group, participants who engaged in evening eating had improved diet quality, suggesting that late eating did not impact their diet quality and that meal timing may not be as relevant for young adults who already engage in healthy dietary patterns. However, for the average young adult, interventions geared towards modifying evening food choices may be more effective at improving diet quality.

### Electronic Supplementary Material

Below is the link to the electronic supplementary material.

Supplementary Material 1

## Data Availability

The data presented in this study are available on request from the corresponding author subject to ethical approval.
