# Supplementary material for: The association between diet quality and chrononutritional patterns in young adults
Source: Eur J Nutr. 2024 Feb 22;63(4):1271–81. doi: 10.1007/s00394-024-03353-7 (PMC11139707; doi:10.1007/s00394-024-03353-7)
Supplement: Supplementary file 1 — Supplementary Material 1 [file 394_2024_3353_MOESM1_ESM.pdf]

SUPPLEMENTARY MATERIAL

Article title: The association between diet quality and chrononutritional patterns in young adults
Journal name: European Journal of Nutrition

Author names: Leanne Wang[1,2], Virginia Chan[1,2] , Margaret Allman-Farinelli[1,2], Alyse Davies[1,2], Lyndal Wellard-Cole[1,3], Anna Rangan[1,2]
Affiliation: [1]Discipline of Nutrition and Dietetics, Susan Wakil School of Nursing and Midwifery, Faculty of Medicine and Health, The University of Sydney, Sydney, NSW 2006, Australia
[2]Charles Perkins Centre, The University of Sydney, Sydney, NSW 2006, Australia
[3]Cancer Prevention and Advocacy Division, Cancer Council NSW, Sydney, NSW 2011, Australia
E-mail address of the corresponding author: anna.rangan@sydney.edu.au

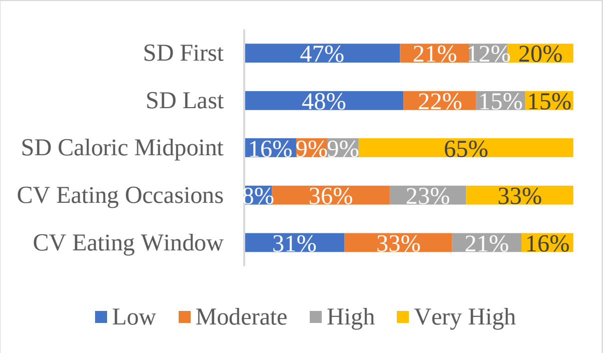

Fig. S1 Stacked bar chart showing the percentage of all participants in the low, moderate, high, and very high meal timing variability categories for each metric over the 3 days of data collection.

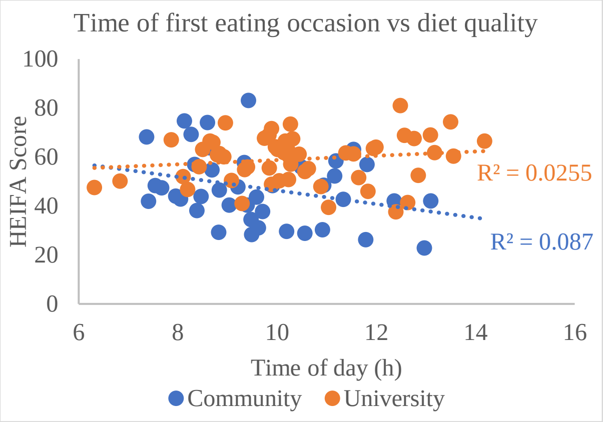

Fig. S2 Scatter plot with line of best fit showing the relationship between each participant's average overall diet quality score with their average time of first eating occasion.

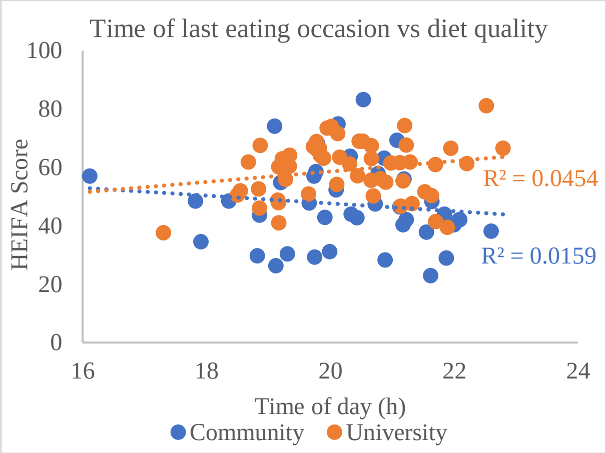

Fig. S3 Scatter plot with line of best fit showing the relationship between each participant's average overall diet quality score with their average time of last eating occasion.

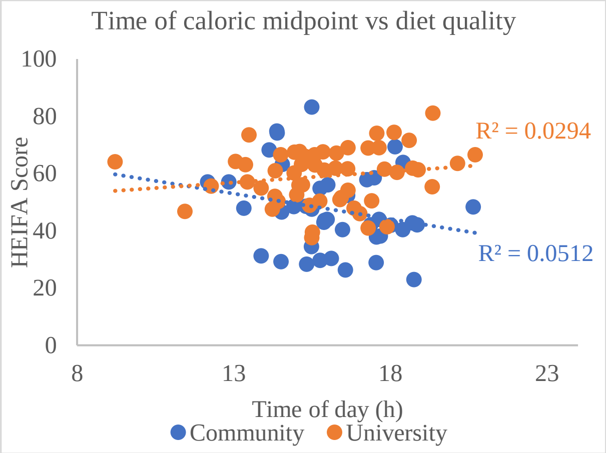

Fig. S4 Scatter plot with line of best fit showing the relationship between each participant's average overall diet quality score with their average caloric midpoint.

Table S1 Associations between chrononutritional variables and diet quality of university and community samples after adjusting for gender, body mass index, and socio-economic status[a]

	University sample (*n* = 54)								Community sample (*n* = 41)							
	Overall Diet Quality		Discretionary		Total Veg		Total Fruit		Overall Diet Quality		Discretionary		Total Veg		Total Fruit	
	β	*p*	β	*p*	β	*p*	β	*p*	β	*p*	β	*p*	β	*p*	β	*p*
Time of first eating occasion	0.036	0.173	0.285	<0.001	-0.115	0.337	-0.002	0.989	-0.382	0.443	0.184	0.605	-0.440	0.072	-0.797	0.040
Time of last eating occasion	0.016	0.302	0.018	0.706	0.012	0.870	0.036	0.681	0.833	0.070	-0.445	0.298	0.166	0.581	0.146	0.761
Caloric midpoint	0.026	0.443	0.148	0.158	-0.023	0.884	-0.037	0.848	-0.034	0.118	-0.143	0.115	-0.031	0.815	-0.045	0.589
Number of eating occasions per day	-0.013	0.475	-0.190	<0.001	0.132	0.094	0.181	0.059	1.725	0.302	-0.722	0.063	0.604	0.024*	0.153	0.730
Daily eating window	-0.020	0.466	-0.267	<0.001	0.127	0.126	0.038	0.802	2.007	0.087	-0.307	0.273	0.347	0.073	0.562	0.069
SD First	0.003	0.788	0.017	0.676	0.097	0.093	0.025	0.724	-2.065	0.220	-0.049	0.904	-0.341	0.221	0.131	0.771
SD Last	0.008	0.525	0.054	0.178	-0.056	0.347	0.066	0.369	-1.149	0.656	0.784	0.193	-0.613	0.145	0.012	0.985
SD Caloric Midpoint	0.499	0.604	0.610	0.069	0.117	0.442	-0.180	0.315	-1.101	0.471	-0.263	0.466	0.281	0.316	-0.017	0.967
CV No. of Eating Occasions	0.000	0.907	0.009	0.256	-0.002	0.885	0.019	0.176	-4.032	0.794	3.614	0.317	-1.223	0.630	-4.533	0.261
CV Eating Window	0.004	0.094	0.018	0.026	0.015	0.196	0.032	0.026	-15.886	0.134	1.949	0.442	-4.264	0.012*	-1.722	0.545

Significance at *p* value ≤0.05

[a]Socio-economic status was determined using the Socio-Economic Indexes for Areas 2016 Index of Relative Socio-economic Advantage and Disadvantage [33] based on their postcode.
